# A Cross-Sectional Comparative Study of Sleep Disturbances in Children with ADHD and Matched Controls

**DOI:** 10.3390/brainsci12091158

**Published:** 2022-08-30

**Authors:** Angela Ann Joseph, Anupama Gupta, Nandita Hazari, Mani Kalaivani, Ravindra Mohan Pandey, Rajesh Sagar, Manju Mehta, Garima Shukla

**Affiliations:** 1 Jindal School of Liberal Arts and Humanities, Institution of Eminence—O.P. Jindal Global University, Sonipat 131001, Haryana, India; 2 Department of Psychiatry, All India Institute of Medical Sciences, New Delhi 110029, Delhi, India; 3 Department of Neurology, All India Institute of Medical Sciences, New Delhi 110029, Delhi, India; 4 Vidyasagar Institute of Mental Health and Neuro Allied Sciences, New Delhi 110029, Delhi, India; 5 Department of Biostatistics, All India Institute of Medical Sciences, New Delhi 110029, Delhi, India; 6 Department of Medicine, Queens University, Kingston, K7L 2V7 ON, Canada

**Keywords:** sleep disturbance, ADHD, polysomnography

## Abstract

Background: Systematic reviews conducted on sleep disturbances in attention deficit hyperactivity disorder (ADHD) have found inconsistent results due to the presence of several moderating variables which were not controlled for in previous studies. The aim of this study was to examine sleep disturbances in children with ADHD compared to their typically developing peers after controlling for moderating variables (age, sex, medication status, body mass index, and psychiatric and medical comorbidities). Methods: ADHD was diagnosed using DSM-IV-TR criteria (Diagnostic and Statistical Manual of Mental Disorders) and Conners’ Parent Rating Scales. Children recruited (aged 6–12 years) for the ADHD group (*n* = 40) met the following criteria: IQ > 80, unmedicated, and no psychiatric or medical comorbidities. The control group consisted of age- and sex-matched typically developing peers (*n* = 40). Sleep was assessed subjectively (through parent reported questionnaires and sleep logs) and objectively (using video polysomnography). Results: 65% of children with ADHD had a sleep disorder, as compared to 17% of controls. The ADHD group reported more sleep disturbances and disorders, both on subjective measures and objective measures. Conclusions: Sleep disturbances and primary sleep disorders in children with ADHD exist independent of moderating variables and differences in sleep assessment methods, thereby bolstering support for previously documented literature on the ADHD and sleep connection.

## 1. Introduction

Attention deficit hyperactivity disorder (ADHD) is one of the most common neurodevelopmental disorders in childhood. It is characterized by symptoms of inattention, hyperactivity, or both, and its global prevalence estimate is 84.7 million cases worldwide [1]. ADHD has an adverse impact on emotional regulation, impulse control, social skills, and academic and occupational functioning [2,3,4]. Therefore, it is associated with a high global burden of disease that places a significant cost on public mental health and quality of life [5].

Sleep problems often co-occur in children with ADHD. About 50–70% of these children reportedly suffer from sleep impairments. Noradrenergic and dopaminergic neurotransmitter pathways have been implicated in both ADHD and sleep disorders [6] and the nature of the relationship between them is considered bidirectional [7]. It has been noted that symptoms of ADHD, such as inattention, hyperactivity, and irritability, may mimic daytime symptoms of primary sleep disorders, and sleep disorders may exacerbate ADHD symptoms [8]. Thus, there exists the possibility that sleep disorders are misdiagnosed as ADHD and vice versa [9]. This has implications for treatment outcomes if the child is prescribed stimulant medication before the sleep impairment is identified, thereby worsening the sleep problem [10]. Moreover, sleep problems in ADHD may affect quality of life, caregiver mental health, emotional self-regulation, and academic, cognitive, and family functioning [11]. Early identification of sleep disturbances is, therefore, essential for effective treatment planning of those with ADHD.

Sleep disturbances associated with ADHD include long sleep latency, delayed sleep phase syndrome, increased periodic limb movement in sleep, daytime sleepiness, altered total sleep duration, restless sleep, and difficulty initiating and maintaining sleep [12,13]. However, systematic reviews and meta-analysis of sleep problems in children with ADHD have reported diverse and inconsistent results [14]. This is partially due to the heterogeneity of studies using different methods of sleep assessment (subjective versus objective measures), methodological limitations (using only clinical samples, no control group, and lack of an adaption night), and not controlling for the presence of moderating variables that influence the relationship between sleep and ADHD [14,15]. Moderating variables include increased susceptibility to inadequate sleep hygiene, use of psychostimulants, comorbid psychiatric conditions, elements intrinsic to ADHD, and medical disorders [16]. Therefore, there is a need for more robust studies that re-examine the topic by taking into consideration the aforementioned factors to provide clarity into the nature and frequency of sleep disorders [14].

The present study is an attempt to address this gap in the literature by re-examining the connection between ADHD and sleep using a rigorous method of inquiry as recommended by previous meta-analysis [15]. We aimed at achieving this by controlling for moderating variables (age, sex, medication status, BMI, IQ, and psychiatric and medical comorbidities) and incorporating a novel methodological approach (using both subjective and objective measures, using standardized diagnostic criteria, and using a community-based control group). Our research intends to add to the available literature by providing a clearer picture on the nature and extent of sleep disturbance in children with ADHD. This, is turn, has clinical implications for accurate evaluation and treatment of sleep problems, which may improve the quality of life and ameliorate ADHD symptoms [17]. The aim of this study was to examine whether there exists a difference in the nature and frequency of sleep disturbances in children with ADHD compared to controls after controlling for moderating variables.

## 2. Methods

This was a prospective case-controlled study enrolling children with ADHD attending the Child and Adolescent Clinic of the Psychiatry out-patients services at our center, with age- and sex-matched controls. The study was conducted between July 2011 and July 2015.

The study population consisted of consecutive children with ADHD and an equal number of typically developing peers within the age range of 6 to 12 years, whose parents/legal guardians consented for participation. Purposive sampling was used and participants were matched for sex and age. Sample size was calculated by comparison between 2 independent proportions to detect the excess absolute difference of 35% in the primary outcome variable—sleep disturbance [Non ADHD group (30%), ADHD group (65%)] at 95% level of confidence and 80% power. It was required to enroll a minimum of 37 children in each of the 2 groups.

### 2.1. Participants

Children with ADHD symptoms present in two settings, with IQ > 80, and who had never been medicated were included in the ADHD group. Those who had comorbid psychiatric or medical conditions (epilepsy or craniofacial abnormalities) or were obese (BMI ≥ 95th percentile) were excluded from the ADHD group. Typically developing children with the absence of psychiatric or medical illness and IQ > 80 were included as controls. Those with psychoactive drug use, medical illness, and BMI values within the obese range were excluded from the control group.

### 2.2. Procedures

Participants were screened for the eligibility criteria and, after obtaining informed consent from the parent/guardian of the child, assessment for psychopathology (via parent-rated and teacher-rated ADHD measures) and sleep disturbances (questionnaires and overnight polysomnography) were conducted.

### 2.3. Behavioral and Cognitive Measures

#### 2.3.1. The Mini International Neuropsychiatric Interview for Children and Adolescents—Parent Version (MINI-Kid)

The Mini International Neuropsychiatric Interview for Children and adolescents—Parent Version (MINI-kid) was used to assess for 23 DSM-IV Axis 1 disorders and rule out the presence of comorbid psychiatric conditions in our participants. MINI-Kid has been utilized in numerous studies to assess Axis I psychopathology in children and adolescents. It has a low cost, short administration time (30 min), and has both parent and child versions, with good concordance between both versions (κ = 0.46–0.94) [18]. The interview schedule has questions inquiring into each of the 23 mental disorders with special screening and assessment items. During the diagnostic interview, which was conducted by a trained mental health professional, parents responded to the interview questions with a “yes/no” so that an appropriate diagnosis could be made.

#### 2.3.2. Malin’s Intelligence Scale for Indian Children (MISIC)

Malin’s Intelligence Scale for Indian Children (MISIC) was used to assess intellectual quotient (IQ) in our participants. MISIC is an Indian adaptation of Wechsler’s Intelligence Scale for Children (WISC). This test is similar to the WISC and includes questions adapted to meet the level of educational and general awareness of Indian children. The MISIC is an individually administered intelligence test for children between the ages 6 and 15 years and generates an IQ score which represents a child’s general cognitive ability. It generates a full-scale IQ which represents overall cognitive ability, which is derived from verbal intelligence quotient (VIQ) and performance intelligence quotient (PIQ). The Indian adaptation established its reliability with the test–retest method and yielded a Pearson’s product moment correlation coefficient at 0.91 for the full-scale IQ. Indian norms are available for ages 6 to 15 years [19].

#### 2.3.3. Conners’ Global Index

ADHD symptoms were examined in two different settings (school and home) using Conners’ Global Index ratings—Teacher Version (CGI-T) and Conners’ Global Index ratings—Parent Version. CGI-T is an assessment tool used to obtain teacher observations about the youth behavior in a school setting (Conners, 1997). It evaluates behavioral problems on 10 items found to be critical in assessing the severity of childhood problems. Teachers are asked to rate these items based on how much the behavior being asked about has been problematic for the past one month. Response options include: never (rated as 0), occasionally (rated as 1), often (rated as 2), and very frequently (rated as 3). Subscales of the test are as follows: restless–impulsive, emotional lability, and total index. Restless–impulsive subscale indicates restlessness, impulsivity, and inability to maintain attention. Emotional lability subscale indicates more proneness to emotional responses/behaviors (crying, anger, etc.) than is typical. Total index reflects hyperactivity and a broad range of general problematic behavior. Raw scores are converted to T-scores by means of a conversion chart depending on the age and sex of the child [20]. Conners’ Global Index ratings—Parent Version were used to characterize patterns of behavior observed by parents at home. The questions, response options, subscales, scoring, and interpretation guideline are similar to the teacher version. Internal consistency was reported as 0.75–0.95 and test–retest reliabilities were 0.71–0.72 [21].

#### 2.3.4. Conners’ Parent Rating Scale—Short Version (Revised)

Clinical assessment of ADHD was conducted using Conners’ Parent Rating Scale—Short Version (Revised). It contains 27 items and can be used for children aged 3 to 17 years. It has four subscales. The subscale labeled “oppositional tendencies” identifies those with oppositional behavior and angry outbursts. The “cognitive problems/inattention” subscale examines academic and organizational difficulties. The hyperactivity subscale looks into behaviors marked by restlessness and impulsivity. The fourth subscale ADHD Index identifies children at high risk for ADHD. Internal reliability coefficient for oppositional, inattentive, and hyperactivity domains is 0.92, 0.94, and 0.91, respectively [22]. 

### 2.4. Assessment of Obesity and Medical Conditions Affecting Sleep

Obesity was assessed using the International Obesity Task Force (IOTF)—Body Mass Index Criteria [23]. BMI was calculated using individual’s weight in kilograms divided by the square of their height in meters. Height was measured by a stadiometer and weight was measured by an electronic weighing machine. Cole and colleagues, as part of the IOTF, proposed age- and sex-specific cut-off points that provide internationally comparable prevalence rates of overweight and obesity in children using data set specific centiles linked to adult cut-off points. Medical conditions affecting sleep, such as craniofacial abnormalities, epilepsy, and others, were ruled out via a clinical examination conducted by a clinician researcher in neurology.

### 2.5. Parent-Reported Sleep Measures

#### 2.5.1. Pediatric Sleep Questionnaire (PSQ)

The Pediatric Sleep Questionnaire (PSQ) [24] was used to assess for sleep-related breathing disorders (OSA). It is a 40-item questionnaire completed by parents and can be used for children within the age range 2–18 years. The responses are rated on a 4-point scale Likert scale (never, rarely, sometimes, and often). It has 5 subscales: (1) OSA, (2) snoring, (3) sleepiness, (4) behavior, and (5) periodic limb movement disorder (PLMD). The PSQ has 0.85 sensitivity and 0.87 specificity. Most of the subscales had fairly good internal consistency coefficients ranging from 0.66 to 0.89, as well as test–retest reliability ranging from 0.62 to 0.92. Internal consistency was 0.71 and test–retest reliability was 0.62 [24]. The PSQ’s OSA scale has been validated in several sleep studies and has clear cut-off scores for referral. In the current study, only the OSA scale (22-item version) was used.

#### 2.5.2. Epworth Sleepiness Scale (ESS)—Modified for Children

The Epworth Sleepiness Scale (ESS)—modified for children was used to assess for excessive daytime sleepiness for use among pediatric populations [25]. The questionnaire contains eight questions that assess the propensity to fall asleep in 8 different situations that most people engage in as part of their daily lives. Items are rated on a 4-point scale (0–3) and internal consistency has been found to be good (Cronbach’s alpha = 0.75). Scores obtained are analyzed using the following categories: getting enough sleep (1–7), tend to be sleepy during the day—this is the average score (8–9), very sleepy and should seek medical advice (10–15), and dangerously sleepy/should seek medical advice (>16).

#### 2.5.3. Child Sleep Habits Questionnaire (CHSQ)

Child Sleep Habits Questionnaire (CHSQ) was used to identify the nature of sleep problems in our study sample. The CSHQ is a parent-reported questionnaire which was developed for children aged 4 to 12 years. It has 45 items which correspond to 6 domains: bedtime resistance, sleep onset delay, sleep duration, sleep anxiety, night waking, parasomnias, sleep disordered breathing, and daytime sleepiness. The CSHQ showed adequate internal consistency for both the community sample (*p* = 0.68) and the clinical sample (*p* = 0.78). Test–retest reliability was acceptable (range 0.62 to 0.79). A cut-off total CSHQ score of 41 yielded a sensitivity of 0.80 and specificity of 0.72 [26]. In the present study, the subscales daytime sleepiness and the sleep disordered breathing were not included, since these two parameters were studied using ESS modified for children and PSQ.

#### 2.5.4. All India Institute of Medical Sciences (AIIMS) Restless Legs Syndrome Questionnaire for Indian Patients

Symptoms of restless legs syndrome (RLS) were examined using the AIIMS RLS Questionnaire for Indian patients [27], which includes the International Restless Legs Syndrome Study Group (IRLSSG) criteria for RLS diagnosis [28] and extensive examination to address socio-cultural variation in RLS presentation. It consists of 9 parts broadly covering demographic details, description of symptoms, topographical distribution, essential IRLSSG diagnostic criteria, supportive symptoms, relieving factors, and others. In adherence to pediatric criteria for RLS, the participant’s report of symptoms in their own words was taken into account along with parental report for arriving at RLS diagnosis. To aid in this process, diagrammatic representation of RLS pain sensations drawn by children were used to help the child understand the questions being asked. Sleep–wake patterns were studied via parent-reported sleep logs that recorded the following parameters for a period of one week: time in bed, time out of bed, total time in bed, time asleep, wake after sleep onset, awake time, total sleep time, and sleep efficiency.

### 2.6. Polysomnography

Sleep was objectively measured using polysomnography (Carefusion, Nicolet One PSG system, San Diego, CA, USA), which is considered the standard in the assessment of sleep. All polysomnography studies were conducted on a Nicolet One long-term monitoring and Sleep system; electrodes were placed according to the international 10–20 system of placing standard electroencephalographic montage (F4-C4, C4-P4, F3-PC3, C3-P3C4-A1, and C3-A2), horizontal electro-oculography, submental and bilateral anterior tibialis electromyography, as per recommendations of the American Academy of Sleep Medicine [29]. Electrocardiography and video recording was conducted using an infrared-light camera. Oronasal airflow was monitored with thermistors. Thoracic and abdominal respiratory movements were recorded using belts with piezo sensors. Respiratory sounds were studied through microphones. Pulse oximetry was used to measure oxyhemoglobin saturation. A nasal cannula was used for recording esophageal pressure. Leg movements were recorded by placement of electrodes over the right tibialis anterior muscle. Sleep stages were visually scored in 30 s epochs, keeping in consideration the rules for pediatric visual scoring as specified by the American Academy of Sleep Medicine [29]. Apnea and hypopneas were scored following AASM definitions to determine the apnea index (AI) and apnea–hypopnea index (AHI) in children. AHI > 2 was considered the criterion for obstructive sleep apnea (OSA) [30]. Periodic limb movements were scored according to standard International Classification of Sleep Disorders-3 criteria, as well as the recommendations from American Academy of Sleep Medicine and the International Restless Legs Study Group [28]. The periodic limb movement index (PLMI) was calculated as the number of periodic limb movements per hour of sleep. PLMI > 5 per hour of sleep was taken as the cut off for periodic limb movement disorder. All participants underwent overnight polysomnography in a sleep lab located in the Neurosciences Center, AIIMS under the supervision of a sleep technician who carried the sleep study and scored the polysomnogram. Interpretation of findings was conducted by an experienced Sleep clinician (AG/GS). One of the parents accompanied the child during the overnight stay at the hospital. Polysomnography was conducted for only a single night, as it was found to be adequate to assess sleep for children with ADHD in the current setting [31].

### 2.7. Data Analysis

#### 2.7.1. Estimation of Sleep Disturbance

Sleep disturbance being a broad concept was broken down to the frequency of sleep problems and sleep disorders. Sleep problems assessed included bedtime resistance, sleep anxiety, sleep duration, night waking, sleep onset delay, and excessive daytime sleepiness. Sleep problems were indicated by a positive endorsement of any item on the CHSQ and a score > 10 on the ESS modified for children. Sleep disorders assessed included OSA, RLS, and PLMD. A score > 0.33 indicated on the PSQ provided subjective data for the presence of OSA. In addition, PSG findings of AHI ≥ 2 were used for objective diagnosis of OSA. RLS was diagnosed clinically using the ARQIP. PLMD was diagnosed using polysomnography.

#### 2.7.2. Statistical Analysis

Statistical analysis was carried out using STATA 11.0 (College Station, TX, USA). Data were presented as number (percentage)/number of pairs or mean ± SD/median (minimum-maximum) as appropriate. All analysis was carried out using statistical methods which were appropriate for matched design, since the controls in the study were age- and sex-matched with the ADHD group. The choice of statistical test was made depending on the nature of the variable (categorical versus continuous) being studied and type of distribution it followed (normal versus non-normal distribution). Generalized estimating equation (GEE) analysis was carried out to compare the difference in the mean values of sleep parameters between groups adjusting for IQ, father’s age, mother’s education, father’s education, and monthly income, since these variables were not balanced between the groups. McNemar’s chi-square test was used to compare the difference in the number of discordant pairs of ADHD and healthy controls to assess for the presence of sleep variables that included OSA and sleep onset delay. Wilcoxon rank sum (Mann–Whitney) test was used to compare the median between the categories of RLS and sleep onset delay within the ADHD group and healthy control group as well. The *p*-value < 0.05 was considered statistically significant.

Ethics clearance from the ethics committee of the All India Institute of Medical Sciences, New Delhi, was taken before commencing the study. Written informed consent was taken from parents/legal guardian of the participants, since the participants were less than 18 years of age. Participation was voluntary and participants had the right to withdraw their participation at any time during the study. Data was kept private and confidential and used for research and publication purposes only.

## 3. Results

### 3.1. Recruitment of Subjects

#### 3.1.1. ADHD Group

**Figure 1:**
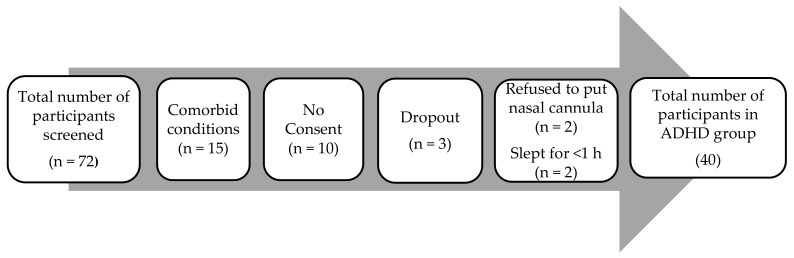
Recruitment process for participants in the ADHD group.

A total number of 72 participants were screened, of which 40 participants completed the study. A total of 32 participants were excluded due to multiple reasons, as depicted in Figure 1. Reasons for exclusion included presence of comorbid conditions, such as conduct disorder (CD), oppositional defiant disorder (ODD), IQ < 80, and seizures. In addition, 10 participants declined consent and 3 voluntarily withdrew their participation after providing consent. Polysomnography data from four participants could not be used for analysis and interpretation due to refusal to wear the nasal cannula and sleeping for less than one hour during the entire duration of the overnight study.

#### 3.1.2. Control Group

**Figure 2:**
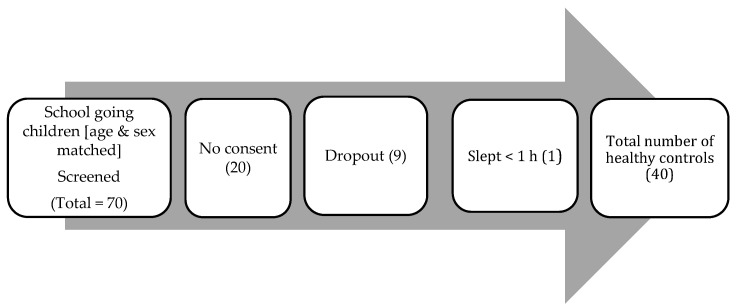
Recruitment process for participants in the control group.

A total of 70 typically developing peers, matched for age and sex with those in the ADHD group, were screened from community for this study. Thirty were excluded for the following reasons: 20 did not consent, 9 withdrew consent, and 1 slept for less than 1 h in the sleep lab (Figure 2). Forty children completed the study.

Hence, the remaining 80 participants (40 in each group) who fulfilled the eligibility criteria and gave their consent for participation were enrolled for this study. These participants were age- and sex-matched across groups. The sex ratio of male: female was 7:1, which was the same in both the groups. Age matching was carried out with a margin of ±1 year. The age (mean ± SD) of children in the ADHD group was 8.4 ± 1.8 years and the age of children in the healthy control group was 8.7 ± 1.6 years. 

### 3.2. Sample Characteristics

Sample characteristics such as age, physical characteristics, parental demographics, and family monthly income were compared between the ADHD and control groups as shown in Table 1.

### 3.3. ADHD Parameters 

#### 3.3.1. Conners’ Global Index (Parent and Teacher Versions)

Parent-reported behavioral measures associated with ADHD were found to be significantly worse in the ADHD group, compared to the control group (Table 2). 

The parent-reported rating for ADHD-related symptoms was higher than teacher-reported symptoms for all behavioral domains assessed on the Conners’ Global Index (Table 3).

Inter-rater agreement between parent and teacher ratings for ADHD symptoms were studied using intra-class correlation coefficients (Table 3). None of these intra-class correlation coefficients between teacher and parent ratings were statistically significant. 

#### 3.3.2. Conners’ Parent Rating Scale—Revised Short Version (CPRS)

Behavioral measures associated with ADHD assessed using Conners’ Parent Rating Scale—Revised Short Version were compared between ADHD and control groups, as shown in Table 4.

ADHD subtypes were characterized using MINI-KID parent version. The combined subtype was the most frequent (*n* = 29, 72%), followed by the inattentive subtype (*n* = 6, 15%), followed by the hyperactive–impulsive subtype (*n* = 5, 13%).

### 3.4. Parent-Reported Sleep History

#### 3.4.1. Pediatric Sleep Questionnaire (PSQ) Findings

Subjective assessment of sleep using PSQ revealed the presence of obstructive sleep apnea (OSA) in 21 out of 40 (52.5%) participants in the ADHD group as compared to 2 out of 40 (5%) participants in the control group (*p* = 0.001), as depicted in Table 5.

An item-wise analysis of PSQ revealed significant differences between ADHD and controls on sleep and behavioral parameters, which included: always snore (*p* = 0.045), snore loudly (*p* = 0.025), mouth breathing (*p* = 0.034), problem with daytime sleepiness (*p* = 0.045), easily distracted (*p* = 0.001), acts as if driven by a motor (*p* = 0.001), does not seem to listen when spoken to directly (*p* = 0.001), difficulty organizing tasks and activities (*p* = 0.001), fidgets (*p* = 0.001), and interrupts others (*p* = 0.001). 

#### 3.4.2. Restless Legs Syndrome Questionnaire Findings

It was found that 12 (30%) children in the ADHD group had RLS, as compared to 3 children (7%) in the control group. The difference in the presence of RLS in two groups was statistically significant (*p* = 0.013), as shown in Table 6.

#### 3.4.3. Epworth Sleepiness Scale—Modified for Children (ESS) Findings

Five children in the ADHD group had excessive daytime sleepiness, as compared to one child in the healthy control group. McNemar chi square test findings revealed that the difference was not statistically significant (*p* = 0.125).

#### 3.4.4. Child Sleep Habits Questionnaire (CSHQ) Findings

Child Sleep Habits Questionnaire was used for assessing sleep problems. Table 7 compares the occurrence of sleep onset delay between the ADHD group and healthy controls. The ADHD group had a greater number of participants with sleep onset delay, as compared to control group, and the difference observed was statistically significant (*p* = 0.003).

Table 8 compares mean ± SD scores on sleep problems assessed by CSHQ. It was observed that the mean ± SD value of bedtime resistance was higher among the ADHD group (12.9 ± 1.9) as compared to controls (11.6 ± 2.5) and the unadjusted difference was statistically significant (*p* = 0.015). When the difference between the mean ± SD values for bedtime resistance between the ADHD group and controls was adjusted for sample characteristics it was found that the difference between the groups was no longer statistically significant (*p* = 0.057). A higher number of participants in the ADHD group compared to controls endorsed items that contributed to the sleep duration subscale of CSHQ, such as “sleeps too little” (*p* = 0.045) and “sleeps the same amount each day” (*p* = 0.007). The mean ± SD value of scores obtained on subscale assessing problems in sleep duration was also higher in the ADHD group (5.2 ± 1.9) as compared to controls (4.1 ± 1.4), and the difference was statistically significant before (*p* = 0.003) and after adjustment for sample characteristics (*p* = 0.001). Items pertaining to the subscale assessing parasomnias, such as “awakens screaming/sweating” (*p* = 0.035) and “alarmed by scary dream” (*p* = 0.035), had greater positive endorsement by the ADHD group as opposed to the healthy control group. The unadjusted difference between ADHD and healthy controls for mean ± SD values on the subscale assessing parasomnias was significant (*p* = 0.037). The ADHD group had more complaints of parasomnias as compared to controls; however, when the difference was adjusted for sample characteristics, this finding did not hold true (*p* = 0.072). None of the differences in mean ± SD scores on CSHQ domains that include sleep anxiety, night waking, and daytime sleepiness between the ADHD group and the healthy controls were statistically significant.

#### 3.4.5. Sleep Log 

Week versus weekend sleep schedules were examined using sleep log for cases and controls (Table 9). The ADHD group went to bed later than controls (*p* = 0.049) on weekdays and they also woke up later (*p* = 0.006). The same trend was observed during weekends as well; the ADHD group went to bed later (*p* = 0.006) and woke up later as compared to controls (*p* = 0.001).

No significant difference was observed between the group on any sleep parameter recorded on sleep logs filled by parents (sleep latency, *p* = 0.552; wake after sleep onset, *p* = 0.396; total time in bed, *p* = 0.500; sleep efficiency, *p* = 0.323; and total sleep time, *p* = 0.642).

### 3.5. Sleep Architecture

#### Polysomnography Data

Table 10 compares objective sleep parameters between the ADHD and control group. Children in the ADHD group spent significantly more time in sleep stage N3 (*p* = 0.033). Sleep efficiency was lower in ADHD group. The median (range) value of sleep latency in the ADHD group (26 (3–118)) was higher than the control group (17 (0–87)). Even though the difference was not statistically significant (*p* = 0.064), the median value of sleep latency in the ADHD group was clinically significant. The median (range) value for periodic limb movements in the ADHD group was 0 (0–108), as compared to 0 (0–38) in the control group, the difference between the groups showing a trend towards statistical significance (*p* = 0.051).

The mean ± SD value of sleep efficiency (see Table 11) was lower in the ADHD group compared to the control group ((83.5 ± 12.8%) vs. (88.3 ± 8.4%)) and the unadjusted difference was significant (*p* = 0.045), which, when adjusted for IQ, father’s age, mother’s education, father’s education, and monthly income using generalized estimating equation, still continued to show a strong trend towards statistical significance (*p* = 0.050). The mean value of total sleep time was less in the ADHD group compared to control group ((399 ± 94 min) and (421 ± 70 min), respectively); however, the difference was not statistically significant (*p* = 0.244).

The distribution of AHI values between groups was compared to examine the trend in the frequency and severity of OSA among participants in both groups (Table 12). AHI > 2 was the criterion used to indicate OSA. It was found that 15 (37.5%) participants in the ADHD group, as compared to 11 (27.5%) participants in the control group, qualified for the objectively measured indicator for OSA. However, the between-group difference for the number of participants who met the OSA criterion was not statistically significant (*p* = 0.474). Even though the number of participants with AHI > 5 was greater in the ADHD group (4, 10%) than that of the control group (1, 2.5%), the difference was not statistically significant across groups (*p* = 0.359).

### 3.6. Estimation of Sleep Disturbances in ADHD Versus Control Group

#### 3.6.1. Estimation of Sleep Problems 

It was found that 31 (77.5%) participants in the ADHD group suffered from sleep problems, as compared to 13 (32.5%) participants in the healthy control group. Upon examining the distribution of specific types of sleep problems, it was found that most participants in the ADHD group faced problems with sleep duration (*n* = 15, 37.5%), followed by sleep onset delay (*n* = 10, 25%) and parasomnias (*n* = 7, 17.5%). The most commonly faced sleep problems among the control group were problems with sleep duration (*n* = 4, 1%) and parasomnias (*n* = 4, 1%).

#### 3.6.2. Estimation of Sleep Disorders

It was found that 26 (65%) participants in the ADHD suffered from sleep disorders, as compared to 7 (17.5%) participants in the control group. OSA could be diagnosed in 21 participants (52.5%), 1 participant (2.5%) was diagnosed with PLMD, and 12 (30%), were diagnosed with RLS, compared to only 3 (7%) in the control group. In the control group, three (7.5%) participants each were diagnosed with RLS and PLMD and two (5%) participants were diagnosed with OSA. In addition, among 10 patients, whose polysomnography studies were later included for detailed REM sleep analysis as part of a larger study [32], 6 demonstrated excessive transient muscle activity and/or sustained muscle activity, suggestive of REM sleep without atonia (RWA), and 3 of these had abnormal behaviors corresponding to the RWA, suggestive of REM sleep behavior disorder.

## 4. Discussion

The objective of this study was to examine whether there exists a difference in the nature and frequency of sleep disturbances in children with ADHD compared to their typically developing peers after controlling for moderating variables (age, sex, medication status, BMI, psychiatric, and medical comorbidities). The results of the current study demonstrate significant differences between the ADHD and control groups on various parent-reported measures of sleep, which included OSA, RLS, sleep onset delay, and sleep duration, using PSQ, the AIIMS RLS questionnaire, and CHSQ. Children with ADHD were also found to significantly differ from their typically developing peers on a variety of behavioral and cognitive measures, including emotional lability, restlessness/impulsivity, oppositional tendencies, inattention, and hyperactivity, as measured by the CGI-T/P and CPRS-Short revised version. Sleep architecture examined via polysomnography revealed that children with ADHD had higher stage N3% and total number of awakenings, as compared to their peers. Lastly, quantitative estimation of sleep disturbances in children with ADHD revealed that 77.5% of children with ADHD had a sleep-related problem, as opposed to 32.5% of controls. Similarly, the presence of sleep disorders was higher among children with ADHD (65%) as compared to children in the control group (17.5%). Our data suggest that sleep disturbance in children with ADHD exist independently of the various moderating factors controlled for in our study. A greater number of subjective sleep-related complaints were observed than what was objectively verified using polysomnography. Moreover, the nature of subjectively measured sleep problems (e.g., sleep onset delay and sleep duration) also differed from objectively observed sleep problems (e.g., night awakenings). When the data obtained from the parent-reported sleep logs (sleep extension on weekends), parent-reported sleep questionnaires (problems of sleep duration), and polysomnography (higher number of night awakenings) were integrated to examine the sleep problems identified, it pointed towards the possible presence of insufficient sleep in those with ADHD. It is suggested that the sleep extension that occurred over the weekend among the ADHD group may be an attempt to overcome the sleep debt accumulated during the week, where wake-up times were restricted by early school start times in India. The subsection that follows discusses the nature and frequency of sleep disturbances in ADHD as documented in our study.

### 4.1. Sleep Disturbances 

#### 4.1.1. Sleep Onset Delay

A meta-analysis of sleep disturbances in children with ADHD [33] indicated that children with ADHD had significantly higher bedtime resistance, more sleep onset difficulties, night waking, difficulties with morning awakenings, and daytime sleepiness on subjective report. With the exception of sleep onset delay, observations on subjective sleep parameters in the current study are similar to those reported in the meta-analysis. Even though ODD was part of the exclusion criteria in our study, we found that, similar to the meta-analysis observations, the ADHD group reported sleep onset delay more frequently than the control group. The difference in median values of sleep latency measured via polysomnography were not statistically significant between the ADHD and control group; however, the median value of sleep latency (26 min) for the ADHD group was clinically significant and indicative of a sleep onset delay (>20 min indicates problems with sleep onset) as compared to 17 min for the control group. A Korean study that examined sleep disturbances in children with ADHD found that, based on subjective evaluation using the CSHQ, the ADHD group was associated with sleep onset delay [34]. This study was similar to our study wherein only unmedicated children within the age range of 7–12 years without any comorbid psychiatric or medical illness were recruited, the ADHD and control group were matched for age and gender, and similar distribution of BMI between groups was maintained. The difference in methodology between our study and the study by Choi and colleagues (2010) was that no information was given on whether the groups were controlled for monthly income and parental demographics, whereas our study found a difference in sleep onset delay between groups after controlling for differences in monthly income and parental demographics, both of which are known to influence sleep quality in children [35]. Sleep onset delay can be attributable to a circadian delayed release of endogenous melatonin, which has been documented in medication-free children with ADHD [36]. Sleep onset insomnia may manifest as bedtime discomfort and bedtime resistance, which could be incorrectly attributed to oppositional symptoms [37], especially when ODD symptoms are not ruled out in children with ADHD. Another important reason for this delay could be the higher percentage of restless legs syndrome in ADHD observed by this study, which is often under-reported, as children may not be able to easily describe their symptoms.

#### 4.1.2. Night-Time Awakenings

In the current study, the scores on night waking did not significantly differ between the groups on subjective assessment; however, on objective assessment, the ADHD group had a significantly higher number of total awakenings at night as compared to the control group. Night-time awakenings in children with ADHD using subjective measures have been reported by several authors [17,33,38]. However, meta-analysis of polysomnography data conducted by Diaz-Roman and colleagues [39] did not report a significant difference in night waking between children with ADHD and controls. Inconsistency in results across studies could be attributed to greater night-to-night variability in sleep among children with ADHD [40]. Moreover, parents may not be aware of awakenings that occur at night if the awakenings are not disruptive to their sleep.

#### 4.1.3. Sleep Apnea 

On parent report through the PSQ, 52% of children with ADHD were identified with OSA compared to 5% of healthy controls. Apart from that, we also found that the ADHD group reported frequent snoring, loud snoring, mouth breathing, and problems with daytime sleepiness, as measured by the PSQ, compared to the control group. This finding is in agreement with several parental report studies that have indicated symptoms of OSA being more frequent in children with ADHD than controls [41,42,43]. On PSG, the median AHI and the number of patients with AHI ≥ 1 were not significantly different between the two groups; the ADHD group had a greater number of participants with AHI ≥ 2 and AHI > 5, but the difference was not statistically significant. A home PSG study also did not report any between-group differences for sleep disordered breathing among ADHD children and controls; however, they did not use nasal cannulas to detect mild apneas in their study [44]. We overcame this limitation by using nasal cannulas to detect for milder forms of apneas and still did not find a statistically significant difference for mean AHI values between ADHD and control groups [34,45]. Conflicting conclusions have been drawn by two meta-analyses, with one supporting the association of OSA and ADHD [33], while the other did not consider OSA as a risk factor for ADHD [15]. As stated earlier, high variability in inclusion criteria, OSA definitions, and methods used are likely responsible for these differences. Since a high percentage of patients with ADHD have symptoms of sleep-related breathing disturbances, there is scope for reviewing criteria for referral for treatment.

#### 4.1.4. Restless Legs Syndrome, PLMD, RBD

Our data showed that RLS was significantly higher in the ADHD group (30%) compared to the controls (7%). In a literature review conducted by Cortese and colleagues [46], it was found that approximately 44% of subjects with ADHD have been found to have RLS or RLS symptoms. Apart from that, other researchers have also found varying percentages of RLS/RLS symptoms in their ADHD sample, with some reporting 6.8% [47], 26.1% [48], or 42.7% [49] with RLS. This broad range of values can be due to methodological differences that can be attributed to use of questionnaires versus IRLSSG criteria, age range of samples, medication status, and presence of comorbid conditions. Nonetheless, it is clear that RLS is highly prevalent in an ADHD population, bearing important management implications.

PLMD could be diagnosed in only one ADHD patient in our study, while it has been found in a high percentage (35%) in a large cohort reported by Frye, et al. [50]. This is clearly attributable to the effect of stimulant and psychoactive medications, which nearly 60% patients were on, in the latter study. 

RBD/RWA was identified among many ADHD patients included in our study. This novel finding has been discussed in detail in a previous publication [32].

#### 4.1.5. Sleep–Wake Schedules

Sleep–wake schedules were examined using sleep logs in our study. It was found that children in the ADHD group went to bed later and woke up later than other kids (both on week and weekends). There was a 24-min difference in bedtimes during the week versus weekend for the ADHD group and a 2-h 18-min difference in the mean values for wake-up times between the week and weekend for the ADHD group. Previous studies have found that weekend total sleep times average 30–60 min more than school night sleep times in 10–14-year-olds, and this difference increases to over 2 h by age 18 [51]. This difference in total sleep times has been interpreted as sleep deprivation during school nights, which is then followed by sleep extension during weekends to make up for the underlying sleep debt. This sleep debt has been attributed to various factors, such as early school start times, academic pressures, and social activities during late afternoon or early evening, and a sleep need that does not decrease with puberty [51]. In the current study, since no data were gathered on school start times, academic pressures, and social activities, no inferences could be made regarding the influence of these variables on week versus weekend sleep schedules. However, the fact that, both in the week as well as weekends, the children in the ADHD group went to bed later than their counterparts suggests that children with ADHD do experience sleep onset delay. 

#### 4.1.6. Overall Sleep Quality

When sleep architecture was compared between both groups in our study it was seen that the ADHD group spent a significantly higher percentage of sleep in stage N3 than controls. Similar to our findings, previous research [52,53] studies examining sleep architecture of children with ADHD found an increase in the percentage of stage N3 of sleep. Increase in stage N3 is associated with the alterations in noradrenaline and dopamine transmission present in children who suffer from ADHD [53]. Lastly, our study conceptualized sleep disturbance into two broad categories, which included sleep problems and primary sleep disorders. It was found that about 77.5% of children with ADHD had sleep problems, as compared to 32.5% of healthy controls. Sleep disorders were present in 65% of children in the ADHD group, as compared to 17% of children in the control group. This finding is in agreement with studies on ADHD and sleep in an Indian population. In a study using parent-reported sleep questionnaires, it was found that sleep problems were present in 65.62% of children with ADHD (ADHD diagnosed based on DSM-IV TR criteria), as compared to 30% of their healthy siblings [54]. This study ruled out organic medical conditions and used unmedicated children, similar to the current study, but did not use standardized rating scales for diagnosis, examine for primary sleep disorders, or rule out psychiatric comorbid conditions. In another study using parent-reported sleep questionnaires and actigraphy, it was found that 60% of children with ADHD and 30% of healthy controls were found to have at least one sleep-related disturbance [55]. This study utilized a clinical sample recruited from psychiatric clinics, used standardized diagnostic criteria based on DSM IV TR and rating scales to assess for ADHD, unmedicated kids with IQ > 80, and ruled out psychiatric comorbid conditions. Controls in this study were age- and gender-matched to the ADHD group. Thus, our research findings are in agreement with studies on ADHD and sleep in an Indian population.

### 4.2. Implications and Strengths of the Study

Overall our data indicate that sleep disturbances may play a pivotal role in the etiology and pathogenesis of ADHD symptoms [56], due to the high prevalence of SRBD, RLS, night wakings, and sleep onset delay that we observed in our ADHD cohort. Previous research has documented that hypoxia and night-time arousals associated with SRBD can affect neurochemical processing in the prefrontal cortex, leading to behavioral and cognitive symptoms associated with ADHD [57,58]. Similarly, RLS is also known to be linked to ADHD via a dopamine deficit common to both disorders associated with the nigrostriatal brain region [59]. Lastly, circadian rhythm sleep disorders may affect ADHD presentations through a behavioral and biological route. A delay in the release of endogenous melatonin and an evening circadian preference have been noted in those with ADHD, as per the previous literature [60]. In addition, it has also been noted that impulsivity and oppositional tendencies manifesting as bedtime struggles may result in delayed sleep onset [61], thus implying that bi-directionality within the ADHD and sleep relationship.

The major strength of our study was in the strict protocol that it followed, which was in adherence to the recommendations provided in the meta-analysis of polysomnographic studies conducted in children with ADHD by Sadeh et al. [15], which is still relevant in current times with regard to the eligibility criteria that Martins et al. [14] used for studies included in their systematic review. Moreover, studying unmedicated patients clearly eliminated medication effect on several sleep metrics. Finally, not only did our study account for the factors mentioned in the eligibility criteria, using both subjective and objective measures, it also accounted for obesity and medical comorbidities, IQ, and matched groups for age and sex. Sadeh, et al. [15] concluded that the heterogeneity of studies made it difficult to integrate measures such that it could be accurately summarized, thereby emphasizing the need for further robust research into sleep parameters associated with ADHD. Our study has managed to do so within certain limitations given the multitude of moderating variables involved. It has provided some clarity on the nature and frequency of sleep disturbances in ADHD, highlighting the need for screening children with ADHD for sleep problems, which may worsen their ADHD symptoms and adversely affect their quality of life if left untreated. Furthermore, insufficient sleep may manifest as impairment in daytime functioning (mimicking symptoms of hyperactivity or inattention) if not corrected.

### 4.3. Generalizability of Findings and Limitations

Certain methodological constraints of our study limited the generalizability of our results. One among them was using a small sample size. There were practical limitations in recruiting a larger sample, especially for the control group, since spending a school night in a sleep lab was inconvenient to children and their parents. Moreover, in adherence to our study center’s research protocol, participants could not be compensated for their participation; hence, there was no incentive provided to encourage participation. Despite these limitations, however, we were able to recruit enough controls for an 80% statistically powered study. The data on night-to-night variability in sleep patterns among those with ADHD have been inconclusive. Some studies report its influence on the presence of sleep disturbances [62,63,64], while others have found that it affects sleep patterns in both ADHD cohorts and their typically developing peers similarly [65]. While we did analyze 13 children with ADHD for a first night effect and did not find any evidence to suggest that sleep on an adaptation night differed from that spent on a second night in a sleep laboratory [31], we did not test for a first night effect on controls and, hence, cannot rule out the limitation that it may have made a difference in the quality of sleep experienced by them in the lab [66]. Another methodological limitation was that we did not use a multiple sleep latency test to objectively assess for excessive daytime sleepiness in our participants. Although we used both objective and subjective measures for sleep disturbances, we found that the nature of sleep disturbances varied between them. Differences observed between subjective and objective measures have been attributed to a variety of factors, which include negative bias in responding to the items, specifically in the context of a difficult parent–child relationship, which leads to over-reporting of symptoms [33,67]. Some researchers suggest that the difference is not due to subjective versus objective measures but rather one that is between prospective and retrospective measures [67]. Subjective measures which are retrospective look at sleep-related behavior, whereas objective measures that are prospective measure sleep physiology and, hence, they yield different results [68].

Recent research conducted in this area has identified several factors that mediate the relationship between sleep and ADHD, some of which are the role of screen time [69,70], sleep hygiene [16], motor proficiency [71], and sensory modulation [72] on sleep in children with ADHD, which we did not account for in our study. Assessing screen time and sleep hygiene would have been especially significant given that our data indicated that those affected by ADHD accumulated sleep debt manifesting in sleep extension over the weekends. A time difference of more than 90 min in sleep duration between week and weekends is an indicator of insufficient sleep [73]. Examining the role of sleep hygiene and screen time as potential precipitating or perpetuating factors for insufficient sleep in our ADHD cohort would have given us valuable insight into the nature of the problem. In addition, obtaining information about sleep chronotypes in our sample by using a Morningness–Eveningness Questionnaire would have helped us understand sleep preferences as they interacted with ADHD symptoms and sleep–wake patterns [74]. Other lifestyle-related factors that mediate the impact of ADHD on sleep, including diet and physical activity [75], were also not assessed in our study. The exceedingly large number of mediating factors that influence the relationship between ADHD and sleep makes it a difficult endeavor to either control or exclude them when analyzing the ADHD–sleep connection in a cross-sectional case–control cohort.

### 4.4. Future Directions

Research in this area has grown rapidly over the last decade and researchers have shifted from examining cross-sectional samples to study correlation between ADHD and sleep to focus more on longitudinal data analysis to study the casual links between sleep and ADHD across the life span [76]. A recent systematic review has stated that it may not be possible to comprehensively study the causal link between sleep and functional outcomes in ADHD given the complexity of the relationship. The authors of the review propose that it may be time to direct research interests towards developing effective interventions for the management of sleep problems in those with ADHD [12]. Future studies adding to the existing body of literature may consider using a multifaceted research approach. This may involve synthesizing information on sleep parameters related to changes in body temperature, sleep–wake cycle hormones (melatonin, adrenocorticotropic hormone, cortisol, and growth hormone), and their connection with circadian cycles, which may yield a holistic view of sleep problems in ADHD. Lastly, studying how cultural aspects of sleep (such as co-sleeping behavior, which is common in India) are associated with sleep disturbances among children with ADHD may help in understanding the manifestation of sleep problems across cultural environments.

## 5. Conclusions

In conclusion, sleep disturbances and primary sleep disorders are significantly more prevalent in children with ADHD, compared to their typically developing peers, though the nature of these impairments varies between subjective versus objective measures. These findings highlight important therapeutic implications targeting sleep in children with ADHD, with targeting sleep disorders prior to initiation of ADHD medications emerging as an important strategy.

## Figures and Tables

**Table 1:** Comparison of physical characteristics, IQ, parental demographics, and family monthly income between ADHD and control group.

Variable	ADHD (*n* = 40)	Control (*n* = 40)	*p*-Value
Age	8.4 ± 1.8	8.7 ± 1.6	0.439
Height (cm)	130.7 ± 13.5	133.6 ± 12.9	0.326
Weight (Kg)	29.5 ± 9.1	31.2 ± 12.2	0.476
BMI (Kg/m^2^)	16.9 ± 3.5	17.0 ± 4.1	0.961
IQ	99.4 ± 10.1	104.3 ± 10.9	0.024 *
Mother’s Age (years)	34.7 ± 4.5	36.7 ± 6.4	0.109
Father’s Age (years)	38.6 ± 4.8	40.8 ± 6.0	0.088
Mother’s Education (years)	13.1 ± 3.4	11.4 ± 4.8	0.073
Father’s Education (years)	13.8 ± 3.5	11.3 ± 4.9	0.016 *
Monthly Income (Rupees)	40,375 (3000–100,000)	29,825 (5000–80,000)	0.079

* *p*-value < 0.05 statistically significant; values have been expressed as mean ± SD or median (Min–Max), paired *t*-test was used to compare means and Wilcoxon signed rank test was used to compare median among the groups. ADHD and normal control groups were matched on age and sex.

**Table 2:** Comparison of Conners’ Global Index—Parent version domains between ADHD and control group.

Domains	ADHD (*n* = 40)	Healthy Control (*n* = 40)	*p* Value
Emotional Lability	77.0 ± 6.7	54.6 ± 6.7	0.001 *
Restless-Impulsive	71.3 ± 11.0	50.3 ± 11.1	0.001 *
Total Index	77.5 ± 7.1	51.5 ± 11.3	0.001 *

Data expressed as mean ± SD, * paired *t*-test was used to compare mean values among groups; *p*-value < 0.05 statistically significant.

**Table 3:** Comparison of Conners’ Global Index—Parent version domains between ADHD and control groups.

Domain	Teacher Rating Mean ± SD (*n* = 38)	Parent Rating Mean ± SD (*n* = 40)	Difference	ICC Value (95% C.I.)	*p* Value
Emotional Lability	69.3 ± 17.1	77.0 ± 6.7	−7.7	−0.05 (−1.02, 0.45)	0.558
Restless Impulsive	64.4 ± 12.0	71.3 ± 11.0	−6.9	0.14 (−0.65, 0.55)	0.319
Total Index	68.5 ± 13.4	77.5 ± 7.1	−9.0	−0.28 (−0.42, 0.20)	0.775

ICC: intra-class correlation coefficient.

**Table 4:** Comparison of Conners’ Parent Rating Scale—Short version (Revised) domains between ADHD and control group.

Domains	ADHD (*n* = 40)	Healthy Control (*n* = 40)	*p* Value
Oppositional	71.1 ± 11.4	47.8 ± 11.1	0.001 *
Cognitive Problems/Inattention	74.0 ± 7.9	46.0 ± 9.6	0.001 *
Hyperactivity	81.9 ± 9.5	52.0 ± 12.3	0.001 *
ADHD Index	81.9 ± 9.5	46.6 ± 9.0	0.001 *

Data expressed as mean ± SD, * paired *t*-test was used to compare mean values among groups; *p*-value < 0.05 statistically significant.

**Table 5:** Comparison of sleep-related breathing disorder in ADHD and control group (ADHD = 40, controls = 40).

SRDB	n_pairs_	*p* Value
ADHD_yes_—Control_yes_	1	0.001 *
ADHD_yes_—Control_no_	20	
ADHD_no_—Control_yes_	1	
ADHD_no_—Control_no_	18	

* Data expressed as number of pairs, * McNemar’s chi square test was used to compare the number of pairs, *p*-value < 0.05 statistically significant.

**Table 6:** Comparison of restless legs syndrome (RLS) between ADHD and control group.

RLS	n_pairs_	*p* Value
ADHD_yes_—Control_yes_	1	0.013 *
ADHD_yes_—Control_no_	11	
ADHD_no_—Control_yes_	2	
ADHD_no_—Control_no_	26	

* Data expressed as number of pairs, * McNemar’s chi square test was used to compare the number of pairs, *p*-value < 0.05 statistically significant.

**Table 7:** Comparison of sleep onset delay between ADHD and control group.

Sleep Onset Delay	n_pairs_	*p* Value
ADHD_yes_—Control_yes_	1	0.003 *
ADHD_yes_—Control_no_	9	
ADHD_no_—Control_yes_	0	
ADHD_no_—Control_no_	30	

Data expressed as number of pairs, * McNemar’s chi square test was used to compare the number of pairs, *p*-value < 0.05 statistically significant.

**Table 8:** Comparison of other subjective sleep parameters between ADHD and control group.

Subjective Sleep Parameters	Mean ± SD		Unadjusted Difference (95% C.I.)	*p* Value	# Adjusted Difference (95% C.I.)	*p* Value
	ADHD (*n* = 40)	Control (*n* = 40)				
Bedtime resistance	12.9 ± 1.9	11.6 ± 2.5	1.4 (0.28, 2.47)	0.015 *	1.2 (−0.03, 2.37)	0.057
Sleep duration	5.2 ± 1.9	4.1 ± 1.4	1.1 (0. 39, 1.85)	0.003 *	1.2 (0.48, 1.89)	0.001 *
Sleep anxiety	3 ± 1.11	3.0 ± 1.2	−0.2 (−0.44, 0.39)	0.905	0.1 (−0.32, 0.62)	0.539
Night awakenings	4.2 ± 1.7	3.9 ± 1.4	0.3 (−0.42, 1.02)	0.407	0.5 (−0.23, 1.21)	0.186
Parasomnias	9.8 ± 3.1	8.5 ± 2.1	1.3 (0.08, 2.51)	0.037 *	1.4 (−0.13, 3.04)	0.072

Data expressed as mean ± SD, * paired *t*-test was used to compare mean values among the groups, *p* value < 0.05 statistically significant. # Adjusted for IQ, father’s age, mother’s education, father’s education, and monthly income using generalized estimating equation.

**Table 9:** Week versus weekend sleep schedule between ADHD and control group.

Sleep Parameters	Mean ± SD		*p* Value
	Cases *n* = 40	Controls *n* = 40	
Bedtime—week	10.18 ± 0.80	9.83 ± 0.922	0.049 *
Wake up time—week	6.46 ± 0.54	6.13 ± 0.56	0.006 *
Bedtime—weekend	10.58 ± 1.71	9.78 ± 1.71	0.029 *
Wake up time—weekend (h)	8.15 ± 1.52	6.43 ± 1.14	0.001 *

Time expressed in hours, data expressed as mean ± SD, * paired *t*-test was used to compare mean values among the groups, *p* value < 0.05 statistically significant.

**Table 10:** Comparison of objective sleep parameters (using polysomnography) between ADHD group and controls.

Objective Sleep Parameters	Median (Range)		*p* Value
	ADHD (*n* = 40)	Control (*n* = 40)	
Apnea–hyponea index	1.5 (0–34.9)	1.3 (0–6.6)	0.732
Sleep latency (min)	26 (3–118)	17 (0–87)	0.064
Sleep time supine (min)	93.7 (0–450)	121.5 (0–427)	0.316
Sleep time lateral (min)	158 (0–377)	142 (0–438)	0.501
Total awakenings	13.5 (1–43)	9.5 (1–59)	0.049 *
Total arousals	47 (0–221)	48 (7–171)	0.590
REM Latency (min)	182.5 (0–316)	160 (0–456)	0.681
Stage W (%)	7.5 (0–53)	7.2 (0–56.8)	0.264
Stage N1 (%)	4 (0–23)	4 (0–54.4)	0.925
Stage N2 (%)	35.5 (1–64)	42.1 (0–73)	0.095
Stage N3 (%)	35.5 (11.2–90)	26.5 (12.1–96)	0.033 *
Stage REM (%)	13 (0–36)	13.5 (0–33)	0.936
Periodic limb movement index	0 (0–17.2)	0 (0–15)	0.149
Arousal index	8.3 (0–32.1)	7.2 (1.1–21.6)	0.500

Data expressed as median (minimum–maximum), * Wilcoxon signed rank test was used to compare median among the groups, *p*-value < 0.05 statistically significant.

**Table 11:** Comparison of objective sleep quality parameters between ADHD and control group.

Objective Sleep Parameters	Mean ± SD		Unadjusted Difference (95% C.I.)	*p* Value	^#^ Adjusted Difference (95% C.I.)	*p* Value
	ADHD (*n* = 40)	Control (*n* = 40)				
Sleep efficiency (%)	83.5 ± 12.8	88.3 ± 8.4	4.9 (0.1, 9.6)	0.045 *	6.6 (0.00, 13.3)	0.050
Total Sleep Time (min)	399.2 ± 93.8	421.2 ± 70.2	19.7 (−61.9, 17.8)	0.270	−27.7 (−74.3, 18.9)	0.244

Data expressed as mean ± SD, * paired *t*-test was used to compare mean values among the groups, *p* value < 0.05 statistically significant, ^#^ adjusted for IQ, father’s age, mother’s education, father’s education, and monthly income using generalized estimating equation.

**Table 12:** Comparison of number of AHI values between ADHD and control group.

AHI Severity	AHI	n_pairs_	*p* Value
AHI > 2	ADHD_yes_—Control_yes_	15	0.474
	ADHD_yes_—Control_no_	25	
	ADHD_no_—Control_yes_	11	
	ADHD_no_—Control_no_	29	
AHI > 5	ADHD_yes_—Control_yes_	4	0.359
	ADHD_yes_—Control_no_	36	
	ADHD_no_—Control_yes_	1	
	ADHD_no_—Control_no_	39	

Data expressed as number of pairs, McNemar’s chi square test was used to compare the number of pairs, *p*-value < 0.05 statistically significant.

## Data Availability

The data presented in this study are available on request from the corresponding author. The data are not publicly available due to privacy and confidentiality concerns.
